# Prevalence and associated factors of uncontrolled blood pressure among hypertensive patients in the rural communities in the central areas in Thailand: A cross-sectional study

**DOI:** 10.1371/journal.pone.0212572

**Published:** 2019-02-19

**Authors:** Suprawee Meelab, Itsara Bunupuradah, Jitrada Suttiruang, Salisa Sakulrojanawong, Nisanat Thongkua, Chattarin Chantawiboonchai, Prim Chirabandhu, Sasanpin Lertthanaporn, Krissada Suwanthip, Chindanai Songsaengthum, Boonyagorn Keattisaksri, Peeranut Trakulsuk, Apichat Pittapun, Naowanit Nata, Ram Rangsin, Boonsub Sakboonyarat

**Affiliations:** 1 Phramongkutklao College of Medicine, Bangkok, Thailand; 2 Department of Internal Medicine, Phramongkutklao College of Medicine, Bangkok, Thailand; 3 Department of Military and Community Medicine, Phramongkutklao College of Medicine, Bangkok, Thailand; University of Mississippi Medical Center, UNITED STATES

## Abstract

**Introduction:**

Hypertension is a common cardiovascular disease at present. Uncontrolled blood pressure leads to further complications including heart attack, stroke and chronic kidney disease. In Thailand, most of the information related to this issue is collected by hospitals or hospital-based organizations rather than at the community level. The objectives of this study were to determine the prevalence of uncontrolled hypertension and to assess the relationship between patient characteristics (socio-behavioral and clinical) and uncontrolled blood pressure among hypertensive patients in the rural communities in the central areas in Thailand.

**Materials and methods:**

This was a cross-sectional study conducted in Na-Yao and Phra-Pleong rural communities of Thailand in 2018 using the total design method. In all, 406 individuals aged ≥18 years were interviewed using structured questionnaires related to demographic information, higher risk behavior, comorbidities and arthrometric measurement. Blood pressure was assessed for all participants. Uncontrolled hypertension was defined as BP ≥140/90 mmHg.

**Results:**

The prevalence of uncontrolled hypertension was 54.4% (males: 59.8%; females: 52.4%). Uncontrolled blood pressure was associated with neck circumference more than 35.75 cm for males and 32.75 cm. for females (adjusted odds ratio; 1.66, 95% confidence interval; 1.03–2.68), sedentary behavior more than 5 hours a day (adjusted odds ratio; 2.03, 95% confidence interval; 1.28–3.23) and missed doctor appointments (adjusted odds ratio; 3.29, 95% confidence interval; 1.09–9.94).

**Conclusion:**

Approximately one half of hypertensive patients in these rural communities had uncontrolled blood pressure. The Ministry of Public Health and health care providers should provide further strategies to prevent uncontrolled blood pressure’s complications.

## Introduction

Nowadays, hypertension is a common and primary risk factor for cardiovascular diseases. [1] It is one of the major global public health issues with the prevalence of 30.8%. [2] The high prevalence of hypertension is reported in Thailand. According to the Thai National Health Examination Survey V (NHES V) conducted in 2015, one in four Thais had hypertension. [3, 4] Hypertension may worsen disease progression and lead to further complications, such as the risk of heart failure, cerebral ischemia, cerebral hemorrhage and chronic kidney diseases. [5–7] Regarding recent available data concerning controlled blood pressure worldwide, the prevalence of controlled blood pressure was 68.9% and 37.5%, in the US and PR China, respectively. [2, 8] In Thailand, the last available information on uncontrolled hypertension from the Thai NHES V in 2015 was defined by systolic blood pressure (SBP) ≥140 mmHg or diastolic blood pressure (DBP) ≥90 mmHg. The report showed that about 62% of Thai hypertensive patients can control their blood pressure. [4] Moreover, almost one half of adult Thais with hypertension were unawareness of high blood pressure. [3, 4] Additionally, uncontrolled blood pressure can establish a broad range of negative outcomes including heart attack, stroke and chronic kidney diseases. [9, 10]

Nevertheless, in Thailand, most information related to hypertension is collected in hospitals or hospital-based organizations rather than at the community level. A half of areas in Thailand are still rural (47%) where the healthcare infrastructure and healthcare provider characteristics differ from those of urban vicinities especially in remote rural areas. [11] Furthermore, the last research related to uncontrolled hypertension was conducted in a rural community in 2012. The report showed that two-thirds patients with hypertension could not achieve the target blood pressure control. [12] Furthermore, the other study in Thailand showed the absolute numbers of individuals with cardiovascular risk factors were greater in rural rather than urban regions. [13] However, only the limited information is available on factors potentially responsible for uncontrolled blood pressure among patients with hypertension in remote rural communities. The required information is essential to focus on preventing the problems. Improving blood pressure control will help to reduce disability from any complications including cardiovascular accident and chronic kidney disease, reduce medical costs and improve quality of life. The present study was conducted directly in a rural population and in primary health care (PHC) units. The objectives of the study were to determine uncontrolled blood pressure and identify risk factors associated with uncontrolled blood pressure among patients with hypertension in the remote rural communities of the central area in Thailand.

## Materials and methods

### Study designs and subjects

This study was conducted in two rural communities of the central areas in Thailand, 160 kms from Bangkok Metropolitan: Na-Yao rural community in Chachoengsao province and Phra-Pleong rural community in Sakaeo province. These remote communities are housing approximately 12,000 people with agriculture as the major occupation. A cross-sectional study was conducted in January 2018. A total survey was used to collect the information from the target population. The registered patients presenting hypertension in these rural communities were retrieved from the databases of PHC units and community hospitals. All of 406 patients with hypertension were interviewed. Inclusion criteria of the study comprised presenting hypertension, receiving medical treatment in a PHC unit or a community hospital for the past 12 months and aged ≥18 years. The patients would have been excluded from the study if they had been pregnant or at their postpartum stage (within eight weeks).

The healthcare system in Thailand can be categorized into two types comprising (1) the healthcare system under the Ministry of Public Health and (2) the private healthcare system such as private clinics and hospitals. All Thais have healthcare coverage schemes such as the universal coverage scheme, social insurance scheme and government officer scheme.

### Data collection

The 420 participants were recruited to the study from PCU officers. Participants were identified by a computerized search of medical records, undertaken by the PCU staff and were asked to participate in the study. Posters in front of the PCU also reminded patients and encouraged them to be interested in participating in the study. The participants visited the PCU to join the study. Moreover, the research team seek for the patients at their houses to encourage them to join the study. From all of 420 patients, 406 hypertensive patients were included in the study. 14 participants were excluded from the study because 2 of them were post-partum women and 12 hypertensive patients did not live in the target areas at the period of studying. The informed consent in Thai was obtained before conducting the research. In case participants could not read the information sheet, the research team would read the information to them and then the participants could use their finger print to confirm their agreement on the consent form. The participants did not receive any incentive for participating in the study. During the study, one participant spent 30 minutes to provide complete information. Face-to-face interviews were conducted by using standardized questionnaires to obtain the required information from patients with hypertension. The interviewers were well trained.

Standardized questionnaires, developed by the research team, covered information on demographics, self-reported comorbidities including diabetes mellitus, dyslipidemia, gout, chronic kidney disease, antihypertensive medication used, physical activities, sedentary behaviors including sitting and sleeping at home, smoking and alcohol consumption. Ex-smoker and Ex-drinker were defined by smoke-free and alcohol-free for 12 months, respectively. Never alcoholic drinking was defined by alcoholic drinking for life-time period. Never smoker were patients who has never smoked, or who has smoked less than 100 cigarettes in his or her lifetime. [14] The sedentary behavior was computed as the average time, in hours per day. The sedentary behavior duration was categorized as: (1) <5 hours/day and (2) ≥5 hours/day. Additionally, self-reported follow-up compliance was investigated including missed doctor appointments. Finally, the use of fish sauce during meals was explored using yes/no questions, because high sodium diets might be harmful for hypertensive patients.

We measured systolic blood pressure (SBP), diastolic blood pressure (DBP), weight (in kilogram) and height (in centimeters), body mass index (BMI) (in kilogram/meter-squared), waist circumference (in centimeters) and neck circumference (in centimeters). Antihypertensive agents were identified and categorized into the following classes: (1) diuretics, (2) anti-adrenergics [α- and ß-blockers], (3) calcium channel blockers [CCBs], (4) angiotensin converting enzyme inhibitors [ACEIs] and (5) angiotensin receptor blockers (ARB).

### Measurements

Hypertension was defined by the Seventh Report of the Joint National Committee on Prevention, Detection, Evaluation and Treatment of High Blood Pressure (JNC 7) hypertension guidelines, i.e., a high blood pressure (SBP ≥140 mmHg or DBP ≥90mmHg). Uncontrolled hypertension was defined by JNC 7 hypertension guidelines, as the hypertensive patients, with medical treatment, have a blood pressure of SBP ≥140 mmHg or DBP≥90 mmHg. [15] Blood pressure was measured by using a mercury sphygmomanometer by an operator trained in standardized technique. The patients were advised to be stationary at least five minutes in a chair, with feet on the floor, and arms supported at the heart level. The patients were asked to avoid caffeine, smoking and exercise for at least 30 minutes before the measurement was taken. Two measurements were taken, and the average was recorded. [15] BMI was calculated as body weight in kilograms divided by height in weight (kg)/height (m)^2^. BMI was classified in five groups including <18.5kg/m^2^ (underweight), 18.5–22.9kg/m^2^ (normal range), 23.0–24.9kg/m^2^ (overweight), 25.0–29.9 kg/m^2^ (obesity I), and ≥30kg/m^2^ (obesity II). [16] Waist circumference was measured at the midway between the lowest rib and the top of the iliac crest; L2-L3 to within 1 mm, with the plastic tape. [17] Waist circumference was classified into two groups including <90 cm. (men) and <80 cm (women) and ≥90 cm (men) and ≥80 cm (women). [16] Neck circumference was measured at the midway between the midcervical spine and midanterior neck, to within 1 mm, with the plastic tape. Among men with a laryngeal prominence (Adam’s apple), the neck was measured just below the prominence. [18] Neck circumference was categorized into two groups, i.e., <35.75 cm (men) and <32.75 cm (women) and ≥35.75 cm (men) and ≥32.75(women). [19]

### Statistical analysis

We checked the collected data by using double-data entry for accuracy and completeness and then coded, entered and analyzed by using IBM SPSS Statistics for Windows, Version 23.0. Frequency distribution of demographic characteristics, behavioral data and comorbidities were calculated to determine descriptive statistics of the sample. Chi-square test was used to compare frequency distribution of categorical variables by strata. Kolmogorov-Smirnov test has been used to assess the normality of the continuous data. Normally distributed continuous data were compared by using Student’s *t*-test. Demographic characteristics, behavioral data and co-morbidity were stratified by sex. Binary logistic regression analysis was used to determine the risk factors associated with uncontrolled hypertension. The magnitude of association was presented as crude odds ratios (ORs) with 95% confidence interval (CI). A p-value less than 0.05 was considered as statistically significant. Differences identified between subgroups of patients are considered hypothesis-generating and require confirmation in independent studies. The multivariate analysis was performed to adjust confounders by using logistic regression analysis with enter method which is a default function of SPSS software for simultaneous adding independent variables in the model. Maximum Likelihood (ML) estimation has been used to obtain beta coefficients from logistic regression analysis.

The final model was Y = -0.424 + β_1_(age in years) + β_2_(gender) + β_3_(community) + β_4_(scheme) + β_5_(occupation) + β_6_(smoking) + β_7_(alcoholic drinking) + β_8_(dyslipidemia) + β_9_(gouty) + β_10_(diabetes mellitus) + β_11_(chronic kidney disease) + β_12_(hypertension duration in years) + β_13_(neck circumference) + β_14_ (fish sauce use) + β_15_(sedentary behavior) + β_16_(missed doctor appointments) + β_17_(lifestyle modification) + β_18_(number of antihypertensive drugs). The multicollinearity was tested. The variables significant in univariate analysis and in established relationship with the outcome were included in multivariate analysis. The Hosmer-Lemeshow goodness-of-fit of the logistic regression models was performed with *p*-value = 0.648.

### Ethics consideration

This study was reviewed and approved by the Royal Thai Army Medical Department Institutional Review Board, reference number R142q/60_Exp. The participants consented in agreement with the WMA Declaration of Helsinki–Ethical principles for medical research involving human subjects.

## Results

### Characteristics of the study participants

The sample size of the study totaled 406 patients with hypertension. Descriptive characteristics of the study sample is presented in Table 1. In all, 178 (43.8%) patients are from the Na-Yao community and 228 (56.2%) patients from the Phra-Pleong community. No significant difference was found in demographic characteristics between both rural communities (Na-Yao and Phra-Pleong) (S1 Table). Most subjects in the study with the highest education level in primary school account for 78.8%. The average age of participants is 63.6 (±11) years (range: 35 to 90). Self-reported average hypertension duration is 7.2 (±6.6) years. The average SBP and DBP of all participants are 138.8±15.6 mmHg and 83.0±10.5 mmHg, respectively. 20.5% of males and 33.3% of female participants have diabetes mellitus. In all, 43.8% of male participants and 60.5% of female participants present dyslipidemia.

**Table 1:** Characteristics of adults with hypertension in the rural community, central Thailand.

Variables	Total (N = 406)	Uncontrolled HT	Controlled HT	*p*-value
	n (%)	n (%)	n (%)	
**Gender**				0.178^b^
Male	112 (27.6)	67 (59.8)	45 (40.2)	
Female	294 (72.4)	154 (52.4)	140 (47.6)	
**Age (years) (mean±SD)**	63.6±11	62.9±11	64.5±11	0.133^c^
**Age group (years)**				0.176^b^
<40	3 (0.7)	2 (66.7)	1 (33.3)	
40–49	40 (9.9)	25 (62.5)	15 (37.5)	
50–59	97 (23.9)	55 (56.7)	42 (43.3)	
60–69	156 (38.4)	78 (50.0)	78 (50.0)	
70–79	75 (18.5)	47 (62.7)	28 37.3)	
≥80	35 (8.6)	14 (40.0)	21 (60.0)	
**Communities**				0.824^b^
Na-Yao	178 (43.8)	98 (55.1)	80 (44.9)	
Phra-Pleong	228 (56.2)	123 (53.9)	105 (46.1)	
**Occupations**				0.653^b^
Agriculture	191 (47)	102 (53.4)	89 (46.6)	
Employee	47 (11.6)	25 (53.2)	22 (46.8)	
Merchant	33 (8.1)	22 (66.7)	11 (33.3)	
Unemployed	8 (2.0)	5 (62.5)	3 (37.5)	
Others	127 (31.3)	67 (52.8)	60 (47.2)	
**Education level**				0.816^b^
Illiterate	67 (16.5)	34 (50.7)	33 (49.3)	
Primary school	320 (78.8)	178 (55.6)	142 (44.4)	
Secindary school	17 (4.2)	8 (47.1)	9 (52.9)	
University	2 (0.5)	1 (50.0)	1 (50.0)	
**Healthcare coverage**				0.480^b^
Universal coverage scheme	385 (94.8)	208 (54)	177 (46)	
^a^Others	21 (5.2)	13 (61.9)	8 (38.1)	
**Smoking**				0.324^b^
Never	339 (83.5)	179 (52.8)	160 (47.2)	
Ex-smoker	28 (6.9)	18 (64.3)	10 (35.7)	
Current smoker	39 (9.6)	24 (61.5)	15 (38.5)	
**Alcoholic drinking**				0.181^b^
Never	292 (71.9)	151 (51.7)	141 (48.3)	
Ex-drinker	48 (11.8)	28 (58.3)	20 (41.7)	
Current drinker	66 (16.3)	42 (63.6)	24 (36.4)	
**Comorbidities**				
Dibetes mellitus	121 (29.8)	123 (54.2)	104 (45.8)	0.836^b^
Dyslipidemia	227 (55.9)	66 (53.7)	57 (46.3)	0.910^b^
Gouty	24 (5.9)	14 (58.3)	10 (41.7)	0.692^b^
Chronic kidney disease	39 (9.6)	21 (53.8)	18 (46.2)	0.662^b^
**Number of antihypertensive drugs**				0.429^b^
No medication	72 (17.7)	42 (58.3)	30 (41.7)	
Monotherapy	225 (55.4)	116 (51.6)	109 (48.4)	
Polytherapy	109 (26.8)	63 (57.8)	46 (42.2)	
**Hypertension duration (years) (mean±SD)**	7.2±6.6	7.0±6.5	7.4±6.8	
**Blood pressure (mmHg)**				
SBP (mean±SD)	138.8±15.6	148.8±13.1	126.8±8.2	<0.001^c^
DBP (mean±SD)	83.0±10.5	87.6±10.9	77.4±6.8	<0.001^c^
**Neck circumference (cm) (mean±SD)**	35.1±3.9	35.6±4.2	34.5±3.2	0.004^c^
**Waist circumference (cm) (mean±SD)**	89.9±11.3	90.9±11.3	88.8±11.2	0.066^c^
**BMI (kg/m**^**2**^**)**				0.271^b^
18.50–22.99	97 (23.9)	48 (49.5)	49 (50.5)	
<18.50	23 (5.7)	9 (39.1)	14 (60.9)	
23.00–24.99	71 (17.5)	41 (57.7)	30 (42.3)	
25.00–29.99	173 (42.6)	96 (55.5)	77 (44.5)	
≥30.00	42 (10.3)	27 (64.3)	15 (35.7)	

SD: Standard Deviation; mmHg: millimeter of mercury; SBP: Systolic Blood Pressure; DBP: Diastolic Blood Pressure; cm: centimeter

^a^Others: Government officer scheme, Social security scheme, and Cash

^b^chi-square test

^c^t-test

### Prevalence of uncontrolled hypertension

**Fig 1:**
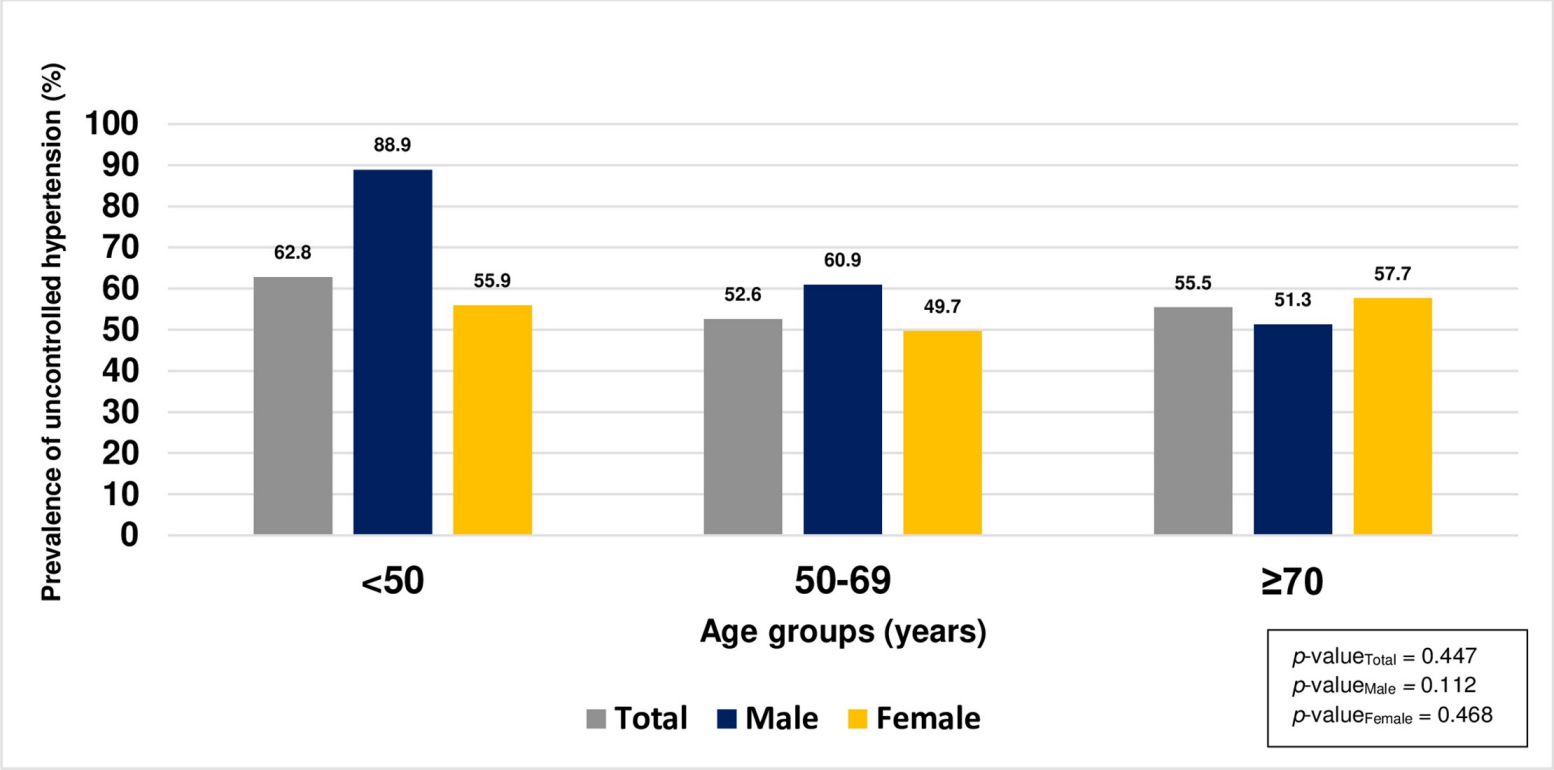
The prevalence of uncontrolled hypertension among adults with hypertension in the rural communities by age and gender.

221 out of 406 participants cannot control blood pressure. Overall prevalence of uncontrolled hypertension in the study was 54.4% (95%CI; 49.6–59.3). Among males and females, uncontrolled hypertension was 59.8% (95%CI; 50.6–69.0) and 52.4% (95%CI; 46.6–58.1), respectively. The prevalence of uncontrolled hypertension in Na-Yao community and in Phra-Pleong community was 55.1% (95%CI; 47.6–62.4) and 53.9% (95%CI; 47.4–60.5) respectively. No significant difference was found in prevalence of uncontrolled hypertension between both rural communities. (Table 2) The prevalence of uncontrolled hypertension in males and in females was no significantly difference by age groups. (*p*-value_male_ = 0.112 and *p*-value_female_ = 0.468) (Fig 1)

**Table 2:** Univariate and multivariate logistic regression analysis to assess relationships between potential risk factors associated and uncontrolled hypertension in the rural communities in the central areas of Thailand.

Variables	Uncontrolled HT	Controlled HT	Crude ORs	95% CI	*p*-value	Adjusted ORs	95% CI	*p*-value
**Age (years)**	62.9±11.0	64.5±11.0	0.99	0.97–1.00	0.133	0.99	0.97–1.02	0.463
**Age group (years)**								
<50	27 (62.8)	16 (37.2)	1.00					
50–59	55 (56.7)	42 (43.3)	0.78	0.06–7.47	0.500			
60–69	78 (50.0)	78 (50.0)	0.59	0.04–5.63	0.139			
70–79	47 (62.7)	28 (37.3)	0.99	0.07–9.68	0.989			
≥80	14 (40.0)	21 (60.0)	0.40	0.03–4.04	0.047			
**Gender**								
Male	67 (59.8)	45 (40.2)	1.00			1		
Female	154 (52.4)	140 (47.6)	0.74	0.48–1.15	0.179	0.95	0.52–1.74	0.867
**Communities**								
Na-Yao	98 (55.1)	80 (44.9)	1.00			1		
Phra-Pleong	123 (53.9)	105 (46.1)	0.96	0.65–1.42	0.842	0.91	0.59–1.42	0.683
**Occupations**								
Agriculture	102 (53.4)	89 (46.6)	1.00			1		
Employee	25 (53.2)	22 (46.8)	0.99	0.52–1.88	0.979	0.84	0.40–1.76	0.647
Merchant	22 (66.7)	11 (33.3)	1.75	0.80–3.80	0.160	1.47	0.64–3.38	0.361
Unemployed	5 (62.5)	3 (37.5)	1.45	0.34–6.26	0.615	1.41	0.28–7.06	0.675
Others	67 (52.8)	60 (47.2)	0.97	0.62–1.53	0.910	0.86	0.49–1.51	0.601
**Healthcare coverage**								
Universal coverage scheme	208 (54.0)	177 (46.0)	1.00			1		
^a^Others	13 (61.9)	8 (38.1)	1.38	0.56–3.41	0.482	1.23	0.43–3.51	0.702
**Smoking**								
Never	179 (52.8)	160 (47.2)	1.00			1		
Ex-smoker	18 (64.3)	10 (35.7)	1.61	0.72–3.59	0.245	1.28	0.46–3.55	0.631
Current smoker	24 (61.5)	15 (38.5)	1.43	0.73–2.82	0.302	1.32	0.54–3.23	0.551
**Alcoholic drinking**								
Never	151 (51.7)	141 (48.3)	1.00			1		
Ex-drinker	28 (58.3)	20 (41.7)	1.31	0.71–2.43	0.395	1.23	0.58–2.63	0.591
Current drinker	42 (63.6)	24 (36.4)	1.63	0.94–2.84	0.081	1.13	0.58–2.2	0.72
**Dyslipidemia**								
No	98 (54.7)	81 (45.3)	1.00			1		
Yes	123 (54.2)	104 (45.8)	0.96	0.63–1.47	0.851	1.06	0.67–1.66	0.813
**Diabetes mellitus**								
No	155 (54.8)	128 (45.2)	1.00			1		
Yes	66 (53.7)	57 (46.3)	0.98	0.66–1.45	0.910	1.01	0.62–1.64	0.976
**Gouty**								
No	207 (54.2)	175 (45.8)	1.00			1		
Yes	14 (58.3)	10 (41.7)	1.18	0.51–2.73	0.693	1.19	0.46–3.03	0.722
**Chronic kidney disease**								
No	200 (54.5)	167 (45.5)	1.00			1		
Yes	21 (53.8)	18 (46.2)	0.97	0.50–1.89	0.938	1.01	0.50–2.04	0.991
**Hypertension duration (years)**	7±6.5	7.4±6.8	0.99	0.96–1.02	0.490	0.99	0.95–1.02	0.454
**Neck circumference (cm)**								
<35.75 in male, <32.75 in female	53 (43.4)	69 (56.6)	1.00			1		
≥35.75 in male, ≥32.75 in female	168 (59.2)	116 (40.8)	1.89	1.23–2.90	0.004	1.66	1.03–2.68	0.037
**Waist circumference (cm)**								
<90 in male, <80 in female	55 (51.9)	51 (48.1)	1.00					
≥90 in male, ≥80 in female	166 (55.3)	134 (44.7)	1.15	0.74–1.79	0.540			
**BMI (kg/m**^**2**^**)**								
18.50–22.99	48 (49.5)	49 (50.5)	1.00					
<18.50	9 (39.1)	14 (60.9)	0.66	0.26–1.66	0.373			
23.00–24.99	41 (57.7)	30 (42.3)	1.40	0.75–2.59	0.290			
25.00–29.99	96 (55.5)	77 (44.5)	1.27	0.77–2.10	0.343			
≥30.00	27 (64.3)	15 (35.7)	1.84	0.87–3.88	0.110			
**Adding fish sauce**								
No	9 (36)	16 (64)	1.00			1		
Yes	212 (55.6)	169 (44.4)	2.23	0.96–5.17	0.062	1.85	0.74–4.59	0.187
**Sedentary behavior (hr.)**								
< 5	116 (48.5)	123 (51.5)	1.00			1		
≥ 5	105 (62.9)	62 (37.1)	1.70	1.20–2.69	0.004	2.03	1.28–3.23	0.003
**Missed doctor appointment**								
No	193 (52.4)	175 (47.6)	1.00			1		
Yes	21 (80.8)	5 (19.2)	3.81	1.41–10.32	0.009	3.29	1.09–9.94	0.035
**Life style modification**								
No	98 (59.8)	66 (40.2)	1.00			1		
^b^Dietary control	33 (55.0)	27 (45.0)	0.82	0.45–1.50	0.523	0.88	0.45–1.72	0.709
^c^Exercise	30 (50.0)	30 (50.0)	0.67	0.37–1.22	0.193	0.77	0.40–1.51	0.447
Dietary control and Exercise	60 (49.2)	62 (50.8)	0.65	0.41–1.05	0.076	0.79	0.47–1.36	0.400
**Number of antihypertensive drugs**								
No medication	42 (58.3)	30 (41.7)	1.00			1		
Monotherapy	116 (51.6)	109 (48.4)	0.76	0.45–1.30	0.316	0.97	0.51–1.86	0.924
Polytherapy	63 (57.8)	46 (42.2)	0.98	0.54–1.79	0.943	1.35	0.66–2.76	0.410

^a^ Others: Government officer scheme, Social security scheme, and Cash

^b^ Dietary control: restricting to only half table spoon of salt per day.

^c^ Exercise: aerobic exercise for 30 minute per day and 3 days per week

BMI: body mass index; Cm: centimeter; kg/m^2^: Kilogram/meter^2^; hr: hour; ORs: odds ratio; CI: confidence interval

**Multivariate logistic regression analysis (Enter): adjusted for** age, gender, communities, occupations, healthcare coverage, dyslipidemia, diabetes mellitus, gouty, chronic kidney disease, hypertension duration, (variables of interest; smoking, alcoholic drinking neck circumference, adding fish sauce, sedentary behavior, missed doctor appointment, life style modification and number of antihypertensive drugs).

### Associated factors of uncontrolled hypertension

Univariate and multivariate logistic regression analysis were performed to determine the factors associated with uncontrolled hypertension (Table 2). After adjusting for the potential confounders, the risk factors associated with uncontrolled hypertension were an increase in neck circumference (Adjusted odds ratio (AORs); 1.66, 95%CI; 1.03–2.68), the sedentary behavior over 5 hours/day (AORs; 2.03, 95%CI; 1.28–3.23) and missed doctor appointments (AORs; 3.29, 95%CI; 1.09–9.94).

## Discussion

This study found the high prevalence of uncontrolled hypertension in the rural communities. The risk factors for uncontrolled hypertension comprised an increase in neck circumference of patients with hypertension, the length of time spent on sedentary behavior and missed doctor appointments. The present study has shown that the prevalence of uncontrolled hypertension among patients with hypertension was 54.4%, lower than that in a related study conducted in the Thai rural community (61.6%). [12] Thai guidelines concerning the treatment of hypertension were established in 2012 and revised in 2015, which was updated by following with American Heart Association and European Society of Cardiology, and then approved by Thai Hypertension Society. Moreover, the revised 2015 version provided behavioral modification for the patients with hypertension including body weight control, aerobic exercise, limitation of sodium intake and dietary approach to stop hypertension (DASH). Therefore, Thai patients with hypertensive have been receiving the appropriate medical treatment. Consequently, the patients with hypertension, who follow-up with their physician, obtain more proper medical care. The prevalence of uncontrolled blood pressure in the hypertensive patients was comparable with the other previous report in rural community in South-Asia developing countries. [20] However, the prevalence was relatively higher than that in 2015 Thai NHES V accounting for 35.1%. [4] The explanation for the difference of the prevalence between the present study and the Thai NHES V is the higher proportion of hypertensive patients without the medical treatment accounting for 17.7% in the study when compared with 6.1% in the Thai NHES V. [4] The hypertensive patients in both rural communities may possess misperceptions and lack knowledge on hypertension, resulting in the high prevalence of continued uncontrolled hypertension. Although, the prevalence of uncontrolled blood pressure in both rural communities was lower than that of the related study conducted in the rural community [12]; however, uncontrolled hypertension could contribute to many complications including heart diseases, cerebrovascular diseases and chronic kidney diseases. [9, 10] This is especially true for patients with hypertension in rural communities, remote from the tertiary health care. Therefore, blood pressure should be controlled to prevent complications involving difficulty accessing tertiary health care far from the community.

The present study found that patients with hypertension, having a neck circumference more than 35.75 cm (in males) and 32.75 cm (in females), are more likely to increase the risk of uncontrolled blood pressure significantly. The similar information was reported in Chinese studies about the relationship between neck circumference and hypertension. [19, 21] Moreover, a study in Israel showed a significant association between relative changes in SBP, DBP by neck circumference. [22] The patient with larger neck circumference may be affected by subcutaneous fat accumulation. Several studies have suggested that fatty composition might affect the level of blood pressure resulting in uncontrolled blood pressure. Various mechanisms including releasing systemic free fatty acid may promote insulin resistance and endothelial dysfunction. [23, 24] In addition, adipocytes are substrates for producing leptin, resulting in elevated leptin levels, leading to the sympathetic nerve activity. [25, 26] Consequently, the sympathetic over activity is induced, leading to increased blood pressure. [27] Moreover, neck circumference is a predictor for obstructive sleep apnea (OSA) [28], contributing to increased blood pressure by neural circulatory mechanisms such as sympathetic activation leading to increased catecholamine levels. [29–31] The previous case control study found that high neck circumference was significantly associated with the risk of high blood pressure, in addition, the combinations of high neck circumference with obesity/abdominal obesity was associated with an elevated blood pressure. The patients with both high neck circumference and obesity/abdominal obesity are more likely to present high blood pressure than those with only one characteristic above. [32] To conclude, neck circumference may be used independently to predict cardiometabolic risks.

The hypertensive patients with sedentary behavior for more than 5 hours a day had increased risks for uncontrolled blood pressure. Similarly, a related cohort study in Spain found that noninteractive sedentary behavior more than 7.9 hours a day was directly associated with a higher risk of hypertension. [33] The study among patients with hypertension in Brazil reported that less time spent in sedentary activities was associated with lower blood pressure. [34] Additionally, one study showed that sitting more than 10 hours a day compared with sitting less than 5 hours a day was associated with increased overall cardiovascular disease risks. [35] This mechanism could explain that the sedentary behavior was linked to metabolic syndrome including hypertension, suggesting increased body weight may result from the sedentary behavior. Obesity generally decreases parasympathetic tone and increases the sympathetic activity, consequently, increasing blood pressure. [33, 36–38]

26 (5.2%) out of 406 hypertensive patients missed doctor appointments. The study found that missing doctor appointments exhibited a significant association with uncontrolled blood pressure. We used subgroup analysis for factors regarding missing doctor appointments. We found that the patients concerning about the side effects of anti-hypertensive medication and believing that medicine was not effective for the treatment are more likely to miss doctor appointment. Moreover, some patients with hypertension believed that they had been cured of the disease already, which is the reason why they lost follow-up with their physicians. All of the subjects possessed misperceptions regarding the knowledge and understanding of hypertension treatment and the lack of awareness of the complications of uncontrolled blood pressure. The study by Knight EL et al., found the lack of knowledge by the patients regarding their target SBP was a dependent predictor of poorly controlled blood pressure. [39]

The study employed a cross-sectional design, which made it difficult to establish a cause-and-effect relationship between associated factors and uncontrolled hypertension. Our findings suggested that modifiable risk factors for uncontrolled blood pressure should be improved. Patients with hypertension especially those residing in rural communities should be targeted for more educational interventions in raising awareness about hypertension, its complication and treatment. Moreover, this finding implied that screening for and managing multiple risk factors should be considered. Nevertheless, the potential of multicollinearity may against the overall fit of the model. The multicollinearity may result due to population unrepresentative sample or insufficient information in the sample. [40] Our study may not be generalized to the whole country, but may reflect challenges of the patients residing in the rural communities of Thailand. Finally, social desirability bias might also exist in the study due to face-to-face interview. However, the interviewers were the well-trained ones.

## Conclusion

In the study, approximately one half of the patients with hypertension residing in rural communities in the central areas in Thailand have had uncontrolled blood pressure. Neck circumference may be used to independently predict uncontrolled blood pressure. In addition, the sedentary behavior more than 5 hours a day may increase the risk for uncontrolled hypertension. Moreover, patients missed the doctor appointments resulting from their misperceptions and the lack of awareness about hypertension complications and treatment. Further, residing in a rural community may reflect poor access to health literacy, so the Ministry of Public Health and health care providers should develop comprehensive guidelines and strategies to allow the hypertensive patients to have access to the standard healthcare system and be educated on hypertension and its complications.

## Supporting information

S1 Table Characteristics of adults with hypertension in the rural community stratified by community of patients, central Thailand. (DOCX)
