# Supplementary material for: Prevalence and associated factors of uncontrolled blood pressure among hypertensive patients in the rural communities in the central areas in Thailand: A cross-sectional study
Source: PLoS One. 2019 Feb 19;14(2):e0212572. doi: 10.1371/journal.pone.0212572 (PMC6380583; doi:10.1371/journal.pone.0212572)
Supplement: S1 Table — (DOCX) [file pone.0212572.s001.docx]

| **Variables** | | | **Overall** | **Communities** | | | | ***p*-value** | |
| --- | --- | --- | --- | --- | --- | --- | --- | --- | --- |
|  | | **(N=406)** | | **Na-Yao n (%)** | | **Phra-Pleong n (%)** | |  | |
|  | | **n (%)** | | **178 (43.8)** | | **228 (56.2)** | |  | |
| **Gender** | | |  |  | |  | | 0.841 | |
| Male | | |  | 50 (28.1) | | 62 (27.2) | |  | |
| Female | | |  | 128 (71.9) | | 166 (72.8) | |  | |
| **Age (years) (mean±SD)** | | | 63.6±11 | 63.4±11.1 | | 63.8±11.0 | | 0.715 | |
| **Age group (years)** | | |  |  | |  | | 0.854 | |
| <40 | | | 3 (0.7) | 1 (0.6) | | 2 (0.9) | |  | |
| 40-49 | | | 40 (9.9) | 19 (10.7) | | 21 (9.2) | |  | |
| 50-59 | | | 97 (23.9) | 45 (25.3) | | 52 (22.8) | |  | |
| 60-69 | | | 156 (38.4) | 62 (34.8) | | 94 (41.2) | |  | |
| 70-79 | | | 75 (18.5) | 35 (19.7) | | 40 (17.5) | |  | |
| ≥80 | | | 35 (8.6) | 16 (9.0) | | 19 (8.3) | |  | |
| **Occupations** | | |  |  | |  | | 0.601 | |
| Agriculture | | 191 (47) | | | 91 (51.1) | 100 (43.9) | |  | |
| Employee | | 47 (11.6) | | | 20 (11.2) | 27 (11.8) | |  | |
| Merchant | | 33 (8.1) | | | 15 (8.4) | 18 (7.9) | |  | |
| Unemployed | | 8 (2.0) | | | 3 (1.7) | 5 (2.2) | |  | |
| Others | | 127 (31.3) | | | 49 (27.5) | 78 (34.2) | |  | |
| **Education level** | | |  |  | |  | | 0.798 | |
| Illiterate | | | 67 (16.5) | 33 (18.5) | | 34 (14.9) | |  | |
| Primary school | | | 320 (78.8) | 137 (77.0) | | 183 (80.3) | |  | |
| Secondary school | | | 17 (4.2) | 7 (3.9) | | 10 (4.4) | |  | |
| University | | | 2 (0.5) | 1 (0.6) | | 1 (0.4) | |  | |
| **Healthcare coverage** | | |  |  | |  | |  | |
| Universal coverage scheme | | | 385 (94.8) | 169 (94.9) | | 216 (94.7) | | 0.926 | |
| ^a^Others | | | 21 (5.2) | 9 (5.1) | | 12 (5.3) | |  | |
| **Smoking** | | |  |  | |  | | 0.487 | |
| Never | | | 339 (83.5) | 153 (86) | | 186 (81.6) | |  | |
| Ex-smoker | | | 28 (6.9) | 10 (5.6) | | 18 (7.9) | |  | |
| Current smoker | | | 39 (9.6) | 15 (8.4) | | 24 (10.5) | |  | |
| **Alcoholic drinking** | | |  |  | |  | | 0.589 | |
| Never | | | 292 (71.9) | 129 (72.5) | | 163 (71.5) | |  | |
| Ex-drinker | | | 48 (11.8) | 18 (10.1) | | 30 (13.2) | |  | |
| Current drinker | | | 66 (16.3) | 31 (17.4) | | 35 (15.4) | |  | |
| **Comorbidities** | | |  |  | |  | |  | |
| Diabetes mellitus | | | 121 (29.8) | 45 (25.3) | | 78 (34.2) | | 0.052 | |
| Dyslipidemia | | | 227 (55.9) | 103 (57.9) | | 124 (54.4) | | 0.484 | |
| Gouty | | | 24 (5.9) | 12 (6.7) | | 12 (5.3) | | 0.532 | |
| **Number of antihypertensive drugs** | | | |  | |  | | 0.508 | |
| No medication | | | 72 (17.7) | 28 (15.7) | | 44 (19.3) | |  | |
| Monotherapy | | | 225 (55.4) | 104 (58.4) | | 121 (53.1) | |  | |
| Polytherapy | | | 109 (26.8) | 46 (25.8) | | 63 (27.6) | |  | |
| **Hypertension duration (years)** | | | 7.2±6.6 | 5.9±5.7 | | 8.2±7.1 | | <0.001 | |
| **Blood pressure (mmHg)** | | |  |  | |  | |  | |
| SBP | | | 138.8±15.6 | 138.7±15.5 | | 138.8±15.8 | | 0.992 | |
| DBP | | | 83.0±10.5 | 82.7±10 | | 83.2±10.9 | | 0.661 | |
| **Neck circumference (cm)** | | | 35.1±3.9 | 34.9±3.3 | | 35.3±4.3 | | 0.299 | |
| **Waist circumference (cm)** | | | 89.9±11.3 | 89.8±10.5 | | 90.0±11.9 | | 0.799 | |
| **BMI (kg/m^2^)** | | |  |  | |  | | 0.837 | |
| 18.50-22.99 | | | 97 (23.9) | 43 (24.2) | | 54 (23.7) | |  | |
| <18.50 | | | 23 (5.7) | 8 (4.5) | | 15 (6.6) | |  | |
| 23.00-24.99 | | | 71 (17.5) | 34 (19.1) | | 37 (16.2) | |  | |
| 25.00-29.99 | | | 173 (42.6) | 76 (42.7) | | 97 (42.5) | |  | |
| ≥30.00 | | | 42 (10.3) | 17 (9.6) | | 25 (11.0) | |  | |

SD: Standard Deviation; mmHg: millimeter of mercury; SBP: Systolic Blood Pressure; DBP: Diastolic Blood Pressure; cm: centimeter

^a^Others: Government officer scheme, Social security scheme, and Cash
